# Target Identification of Active Constituents of *Shen Qi Wan* to Treat Kidney *Yang* Deficiency Using Computational Target Fishing and Network Pharmacology

**DOI:** 10.3389/fphar.2019.00650

**Published:** 2019-06-07

**Authors:** Jie Ying Zhang, Chun Lan Hong, Hong Shu Chen, Xiao Jie Zhou, Yu Jia Zhang, Thomas Efferth, Yuan Xiao Yang, Chang Yu Li

**Affiliations:** ^1^Department of Pharmacy, Zhejiang Chinese Medical University, Hangzhou, China; ^2^Department of Pharmaceutical Biology, Institute of Pharmacy and Biochemistry, Johannes Gutenberg University, Mainz, Germany; ^3^The First Affiliated Hospital of Zhejiang Chinese Medical University, Hangzhou, China; ^4^School of Basic Medical Sciences and Forensic Medicine, Hangzhou Medical College, Hangzhou, China

**Keywords:** network pharmacology, gene ontology, potential targets, traditional Chinese medicine, phytotherapy, transcriptomics

## Abstract

**Background:** Kidney *yang* deficiency syndrome (KYDS) is one of the most common syndromes treated with traditional Chinese medicine (TCM) among elderly patients. *Shen Qi Wan* (SQW) has been effectively used in treating various diseases associated with KYDS for hundreds of years. However, due to the complex composition of SQW, the mechanism of action remains unknown.

**Purpose:** To identify the mechanism of the SQW in the treatment of KYDS and determine the molecular targets of SQW.

**Methods:** The potential targets of active ingredients in SQW were predicted using PharmMapper. Gene Ontology (GO) and Kyoto Encyclopedia of Genes and Genomes (KEGG) pathway enrichment analyses were carried out using the Molecule Annotation System (MAS3.0). The protein–protein interaction (PPI) network of these potential targets and “components-targets-pathways” interaction networks were constructed using Cytoscape. We also established a KYDS rat model induced by adenine to investigate the therapeutic effects of SQW. Body weight, rectal temperature, holding power, water intake, urinary output, blood urea nitrogen (BUN), serum creatinine (Scr), adrenocorticotrophic hormone (ACTH), cortisol (CORT), urine total protein (U-TP), and 17-hydroxy-corticosteroid (17-OHCS) were measured. Additionally, the mRNA expression levels of candidates were detected by qPCR.

**Results:** KYDS-caused changes in body weight, rectal temperature, holding power, water intake, urinary output, BUN, Scr, ACTH, CORT, U-TP, and 17-OHCS were corrected to the baseline values after SQW treatment. We selected the top 10 targets of each component and obtained 79 potential targets, which were mainly enriched in the proteolysis, protein binding, transferase activity, T cell receptor signaling pathway, and focal adhesion. *SRC*, *MAPK14*, *HRAS*, *HSP90AA1*, *F2*, *LCK*, *CDK2*, and *MMP9* were identified as targets of SQW in the treatment of KYDS. The administration of SQW significantly suppressed the expression of *SRC*, *HSP90AA1*, *LCK*, and *CDK2* and markedly increased the expression of *MAPK14*,* MMP9*, and *F2*. However, *HRAS* levels remained unchanged.

**Conclusion:** These findings demonstrated that SQW corrected hypothalamic–pituitary–target gland axis disorder in rats caused by KYDS. *SRC, MAPK14*,* HRAS*, *HSP90AA1*, *F2*, *LCK*, *CDK2*, and *MMP9* were determined to the therapeutic target for the further investigation of SQW to ameliorate KYDS.

## Introduction

Kidney *yang* deficiency syndrome (KYDS) is a diagnostic pattern in traditional Chinese medicine (TCM) and was first documented in *Huang Di Nei Jing*, one of the four great classical textbooks of TCM (Nan et al., 2016). KYDS is characterized by warm dysfunction and a metabolic disorder of the body fluid, causing aversion to cold, cold limbs, cold of waist and back, soreness and weakness of waist and knee, tinnitus, fatigue, impairment of hearing, and looseness of teeth (Lu et al., 2011; Tan et al., 2014; Rong et al., 2016; Xiong et al., 2019). Modern studies have indicated that functional disorders with different degrees of hypothalamic–pituitary–target gland axis, including adrenal glands, thyroids, and gonads, are the crucial pathological mechanism leading to KYDS (Lu et al., 2011; Tan et al., 2014; Nan et al., 2016; Zhang et al., 2017; Tang et al., 2018). KYDS can be present in chronic diseases such as rheumatoid arthritis, hypertension, and diabetes, posing a considerable challenge to the medical system. A valid and classic rat model of KYDS has been developed *via* the administration of a high dose of adenine by oral gavage, which precipitates in renal tubules, leading chronic renal failure, and the animals exhibit the clinical characteristics of KYDS.

*Shen Qi Wan* (SQW) is a frequently used Chinese formula described by Zhang Zhongjing in *Synopsis of Prescriptions of the Golden Chamber* (also named *Jin Kui Yao Lue* in Mandarin). It can be traced back to nearly 2,000 years ago in ancient China (Xiong et al., 2015). The SQW formula is based on the combinatorial principle of “emperor-minister-adjuvant-courier” (*jun-chen-zuo-shi* in Chinese) to combine multiple herbs. The *jun* herb of SQW contains *Cinnamomum cassia (L). J.Presl* and *Aconitum carmichaelii Debeaux* to treat the main cause or primary symptoms of KYDS. The *chen* herb of SQW is *Rehmannia glutinosa (Gaertn). DC.*, *Cornus officinalis Siebold & Zucc*., and *Dioscorea oppositifolia L.* assist the *jun* herb to enhance its therapeutic effects and relieve the accompanying symptoms. The *zuo shi* herb includes *Poria cocos (Schw). Wolf*, *Alisma plantago-aquatica L*., and *Paeonia × suffruticosa Andrews* to counteract the possible toxicity or side effects of other herbs and to ensure the absorption of the formula components and help deliver or guide them to the target organs (Qiu, 2007; Yao et al., 2013). For centuries, SQW has been effectively used in treating various diseases associated with KYDS. However, the therapeutic mechanism remains unknown, the complexity of multiple components, multiple targets, and multiple pathways involved in KYDS make it difficult to elucidate using classical pharmacological approaches.

Network pharmacology is a distinctive new approach based on advances in polypharmacology and network biology to shift away from the traditional “one drug, one target” strategy and move toward sub-network targets and systems, providing a more comprehensive understanding of the mechanism, targets, and pathways behind drug action (Tang and Aittokallio, 2014; Poornima et al., 2016). With the combination of the “medicines-targets” network and biological system network, network pharmacology is becoming more widely known and more frequently used in the field of drug research. PharmMapper (http://www.lilab-ecust.cn/pharmmapper/) is a freely accessed web server designed to identify potential target candidates for probe small molecules of interest using pharmacophore mapping approach (Liu et al., 2010). PharmMapper provides deeper insights and scientific evidence for TCM and helps identify potential targets of Chinese herbs and their underlying mechanisms.

In the present study, investigations based on the pharmacology database and previous studies were conducted to investigate the warm yang and the involvement of several compounds of interest. Potential targets of SQW were predicted by reverse docking to analyze the biological information of potential targets and associated pathways using the network pharmacology method. Moreover, we aimed to identify the potential therapeutic target genes and explore the effects of SQW on the mRNA expression levels of the candidate targets to preliminarily discuss the involvement of the candidate targets in KYDS.

## Materials and Methods

### Compound Preparation

To collect the compounds of SQW, we combined the Traditional Chinese Medicine Systems Pharmacology Database (TcmSP^™^, http://lsp.nwu.edu.cn), a unique system pharmacology platform designed for Chinese herbal medicines (Liu et al., 2016) and the review of previous studies (Wang et al., 2016). In addition, we used China National Knowledge Infrastructure (CNKI) and PubMed to obtain information on the modern pharmacology of the compounds in SQW. CAS No. comes from the Chemical Abstract Service (http://www.cas.org/). We finally selected several compounds of each herb in SQW, every compound we chose has various pharmacological effects such as vascular and tracheal relaxation effect; anti-thrombotic, anti-apoptotic, anti-oxidative effects; and anti-inflammatory and immunomodulatory effects.

### Preparation of Mol2 Format Files

Using the software ChemBioDraw Ultra 14.0 (Version 14, PerkinElmer Inc), we transformed the structures of active components into the sdf structure format. Then, we transformed the sdf structure format into mol2 format files using ChemBio3D Ultra 14.0 (Version 14, PerkinElmer Inc) to obtain the corresponding three-dimensional molecular ball-and-stick model.

### Prediction and Screening of Targets

To predict the potential target candidates, we imported the mol2 format files into the freely accessed web server of the target database of pharmacophore PharmaMapper website (http://www.lilab-ecust.cn/pharmmapper/) to perform reverse docking. Subsequently, we employed UniProtKB (http://www.uniprot.org/), which is the central hub for the collection of functional information on proteins, to correct the unstandardized drug target naming by converting the protein names with the species limited to “Homo sapiens” to its official symbol. We selected the top 10 targets of each active component for the subsequent study.

#### Construction of Protein–Protein Interaction Network for Potential Targets

By using the STRING (Version 10.0) database (http://version10.string-db.org//), we identified the direct physical interactions of proteins and their functional interactions (Wu et al., 2016). We uploaded the gene symbols of potential targets and drew a protein–protein interaction (PPI) network graph online to evaluate the interactions among the potential targets. Then, we imported the PPI data in text format into Cytoscape (http://www.cytoscape.org/) to visualize relationships and used its network analyzer plugin to calculate the degree of PPI network.

### Investigation of Biological Information for Potential Targets of SQW

We imported the potential targets into the Bio database (http://bioinfo.capitalbio.com/mas3/ Version 3.37) to perform the analysis for GO and KEGG pathway enrichment and then screened for pathways with a cut-off *p* < 0.05 (Wu et al., 2016).

### Construction of the Component-Target-Pathway Network

Based on the screening of pathways with their corresponding targets and components, we created a component-target-pathway illustration using Cytoscape, which not only applies to visualizing biological pathways and intermolecular interaction networks but also supplies a basic set of features for data integration, analysis, and visualization for complicated network analysis (Liu et al., 2016). In the network, the node stands for the constituents of SQW, chemical components, component targets, and component pathways. These constituents are connected by an edge when a target is a potential target of a compound. With this network, we studied the effects of multiple components, multiple targets, and multiple pathways of SQW, which ameliorates KYDS.

### Animal Study and Sample Collection

The animal study was approved by the Ethics of Committee of Zhejiang Chinese Medical University. Thirty male Wistar rats (250 ± 30 g, animal license no. SCXK-2013-0033) were obtained from the Animal Center of Zhejiang Chinese Medicine University [Laboratory rearing room Permit No. SYXK (Zhejiang) 2013-0184]. All of the animals were housed at 22 ± 2°C with 50–60% relative humidity. A 12 h light/12 h dark cycle was set, and the animals had free access to standard diet and water. All animals were randomly divided into the control group (n = 10), KYDS model group (n = 10), and SQW group (n = 10). In the first 21 consecutive days, the control group was administered normal saline, the model group and the SQW group were administered adenine (Lot: 131203. Shanghai Bo’ao Biological Technology Co., Ltd) at 200 mg/kg per day. From the 22^nd^ day, the control group was administered normal saline, the model group was administered adenine at 200 mg/kg and normal saline *via* gavage after 1 h per day in the next 21 consecutive days, and the SQW group was administered adenine at 200 mg/kg per day and SQW (Lot: 130904. Henan WanXi Pharmaceuticals Co., Ltd) at 3g/kg *via* gavage after 1 h per day for the next 21 consecutive days. The body weight, rectal temperature, and holding power were detected every 4 days. Water intake and urinary output were observed every 7 days. The urine was obtained from metabolism cages and was used to detect the urine total protein (U-TP) by an automatic biochemical analyzer; 17-hydroxy-corticosteroids (17-OHCSs) were measured using ELISA kits (CAS: 14020809, Biovol Technologies Co. Ltd. Shanghai) after adenine administration at day 21. Blood samples were collected from the heart after pentobarbital sodium (45 mg/kg, i.p.) anesthesia, and then the kidney tissues were rapidly excised, quickly frozen in liquid nitrogen, and stored at −80°C to perform the quantitative real-time PCR assays. Serum was separated by centrifugation at 3,000 rpm for 15 min at 4°C after standing for 30 min to detect blood urea nitrogen (BUN) and serum creatinine (Scr) with an automatic biochemical analyzer (Hitachi, Japan); adrenocorticotrophic hormone (ACTH) and cortisol (CORT) were measured using enzyme-linked immunosorbent assay (ELISA) kits (CAS: 140208, 14020807. Biovol Technologies Co. Ltd., Shanghai). Throughout the experimental period, no animals died before the experimental endpoint. Euthanasia was performed under sodium pentobarbital anesthesia followed by cardiac puncture/kidney removal for all animals.

### Quantitative Real-Time PCR Analysis

Total RNA separation and extraction methods were performed according to the instructions of the TaKaRa MiniBEST Universal RNA Extraction Kit (TaKaRa, Clontech). Spectrophotometric measurements at 260/280 nm (Thermo Scientific, USA) were used to determine the purities and concentrations of the total RNA samples. Reverse transcription reactions were performed using 300 ng of RNA with PrimerScript^™^ RT Master Mix (Perfect Real Time) for cDNA. **Table 1** lists the primer sequences. SRC, MAPK14, HRAS, HSP90AA1, F2, LCK, CDK2, and MMP9 gene expression was investigated. The samples were exposed to pre-denaturation at 95°C for 30 s, followed by 40 cycles of denaturation at 95°C for 5 s, and annealing at 60°C for 30 s. The dissolution curve conditions were 65°C for 0.05 s and 95°C for 0.5 s using 5 µL 5× SYBR Green qPCR Mix, 0.4 µL 20 µmol/L forward primer, 0.4 µL 20 µmol/L reverse primer, and 1 µL cDNA. Water was added to achieve a total volume of 10 µL. β-Actin was used as the internal control, and the data were analyzed using the 2^-ΔΔCt^ method. The experiment was repeated three times.

**Table 1:** Primers used for qPCR.

Gene	Forward primer	Reverse primer
*SRC*	AAGCTTAGGTCTGGCATGGT	ATGGGCTTACGGGGTTACAA
*MAPK14*	GCTTACCGATGACCACGTTC	CGTGGCCTCGGAAATACAAG
*HRAS*	GTACTTGGGTGATGGTTACGTT	AGACTTGGCGCTTGTAAAGGA
*HSP90AA1*	GGGGAAATGACAGGGAAGGA	AGCAATTCTCCTGTCAGCCT
*F2*	CACGGCTACGGATGTGTTCTG	ACCCTCAGCACAGTTACCTTC
*LCK*	TTACCTACCCGCGCTCCTGTGTCCC	CTGGGAAGTCAGTGTCAAACCA
*CDK2*	TACTTTGGGAGGCTGAGGTG	TTCCCCTCCCACTGATTTCC
*MMP9*	CCCCGGTACCGAAGGCGAAATGCTTTGCCC	CCCCCTCGAGGGTGAGAACCGAAGCTTCTG
β*-actin*	GCTCTCTTCCAGCCTTCCTT	GGTCTTTACGGATGTCAACG

SRC, proto-oncogene tyrosine-protein kinase SRC; MAPK14, mitogen-activated protein kinase 14; HRAS, GTPase HRas; HSP90AA1, heat shock protein HSP 90*α*; F2, prothrombin; LCK, proto-oncogene tyrosine-protein kinase LCK; CDK2, cell division protein kinase 2; MMP9, matrix metalloproteinase-9.

### Standards and Chemicals

The standards of o-anisaldehyde (purity >96%), higenamine (purity >98%), and coryneine chloride (purity >98%) were purchased from Yuanye Biological Technology Co., Ltd (Shanghai, China). Salsolinol standard (purity >98%) was purchased from Tauto Biotech Co., Ltd (Shanghai, China). And cinnamic acid standard (purity >98%) was from Chinese National Institute for the Control of Pharmaceutical and Biological Products (Beijing, China). HPLC-grade acetonitrile and methanol were purchased from Tedia (Fairfield, USA). HPLC-grade phosphoric acid was supplied from Shanghai Aladdin Bio-Chem Technology Co., Ltd (Shanghai, China). Distilled water was used throughout the study.

### Preparation of Reference and Sample Solutions

A mixed standard solution was obtained by dissolving the five standards (o-anisaldehyde, higenamine, coryneine chloride, salsolinol, and cinnamic acid) in methanol. The final concentration is 2 mg/ml. SQW was ultrasonically extracted by 10-fold volume pure water twice for 30 min each time. The solution was concentrated to 40 mg/ml then filtered by a 0.22-μm Millipore filter. The injection volume was 10 μl in the same.

### Liquid Chromatographic Analysis

Samples were analyzed using the ACQUITY UPLC system (Waters Corp., Milford, MA, USA), equipped with a quaternary pump and a variable wavelength ultraviolet (UV) detector. Elution of analytes was achieved on an Agilent EC-C18 column (100× 3.0 mm, 2.7 *μ*m diameter) at a flow rate of 0.3 ml/min. The mobile phase was acetonitrile (A) and 0.1% phosphoric acid solution (B). The gradient program was as follow: 0–5 min, 0.5–2% A; 5–6 min, 2–30% A; 6–8 min, 30–50% A; 10–12 min, 50–70% A; 12–15 min, 70–0.5% A. The UV detection wavelength was at 208 nm. The column temperature was set at 25°C.

### Statistical Analysis

The SPSS 22.0 statistical software package (SPSS, Chicago, IL, USA) was used for the analysis of variance followed by one-way ANOVA. Data were presented as the mean values ± standard deviation. Statistical significance was considered if *p* < 0.05 was observed.

## Results

### Compound Information

A search of the TcmSP^™^ identified 1,345 items, including 130 in *A. carmichaelii* Debeaux, 200 in *C. cassia* (L.) J.Presl, 151 in *R. glutinosa* (Gaertn.) DC., 452 in *C. officinalis* Siebold & Zucc., 142 in *D. oppositifolia* L., 68 in *P. cocos* (Schw). Wolf, 92 in *A. plantago-aquatica* L., and 110 in *Paeonia* × *suffruticosa* Andrews. Wang et al. (2016), in “an integrated chinmedomics strategy for discovery of effective constituents from traditional herbal medicine,” reported 84 compounds, among which 51 compounds in negative ion mode and 33 compounds in positive ion mode were identified from SQW. Moreover, 20 compounds absorbed into the blood, such as azelaic acid-O-glucuronide, jionoside D, azelaic acid, and poricoic acid, had a strong relationship with the therapeutic effect of SQW on KYDS. Database search and current studies were used to select the chemical components of SQW according to its pharmacological activities, such as a cardiac-stimulating effect, heightened adrenal cortex function, promoting diuresis and detumescence, invigorating spleen and dampness removal, as shown in **Table 2**. Higenamine, coryneine chloride, salsolinol, o-anisaldehyde, cinnamic acid, catalpol, acteoside, loganin, diosgenin, morroniside, pachymic acid, acetophenone, paeoniflorin, alisol A, and alisol B were probably associated with warming yang attributes of SQW, which were used for further study.

**Table 2:** Chemical information for the constituents of SQW.

Medicinal herbs	Compounds	MW	Structure	Composition	CAS no.	Pharmacological activities	References
*Fuzi* *(Aconitum carmichaelii Debeaux)*	Higenamine	271.32	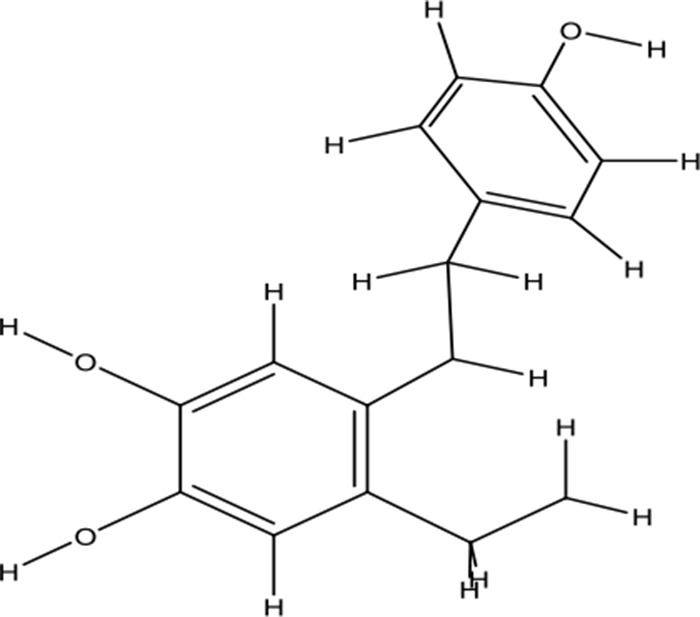	C_16_H_17_NO_3_	5843-65-2	Anti-inflammatory; anti-oxidative; anti-apoptotic	Zhang et al., 2014; Liu et al., 2015; Wei et al., 2015
	Coryneine chloride	203.67	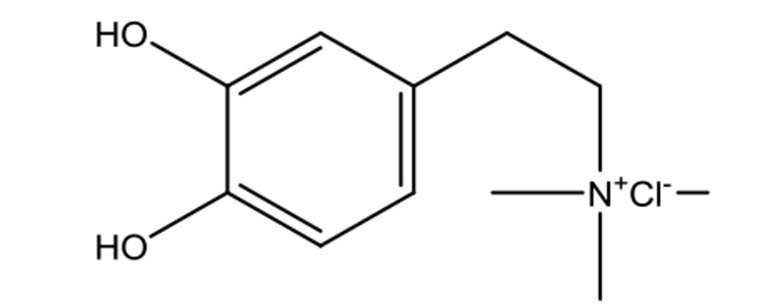	C_9_H_14_ClNO_2_	1477-68-5	Cardiac-stimulating	Li et al., 2010
	Salsolinol	179.22	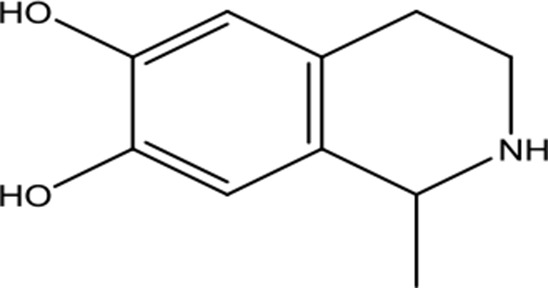	C_10_H_13_NO_3_	27740-96-1	Cardiac-stimulating	Chen and Liang, 1982; Yang et al., 2014
*RouGui* *(Cinnamomum cassia (L.) J.Presl)*	o-Anisaldehyde	162.19	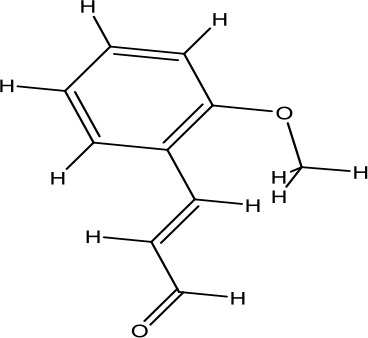	C_10_H_10_O_2_	1504-74-1	Improve blood supply of cardiac muscle; anti-shock	Hasegawa et al., 2002; Chang et al., 2013; Ma et al., 2018
	Cinnamic acid	148.16	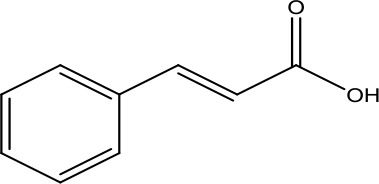	C_9_H_8_O_2_	621-82-9	Protect myocardial function	Li et al., 2019
*Dihuang* *(Rehmannia glutinosa (Gaertn.) DC.)*	Catalpol	362.33	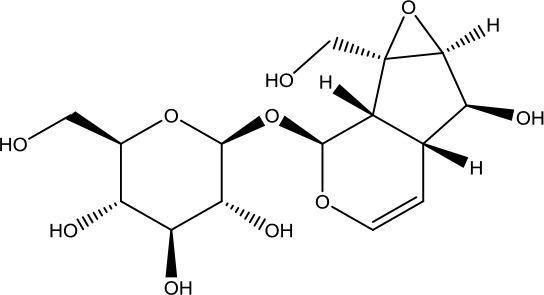	C_15_H_22_O_10_	2415-24-9	Hypoglycemic effect; anti-tumor; anti-inflammatory	Youn et al., 2018; Yuan et al., 2018
	Acteoside	624.59	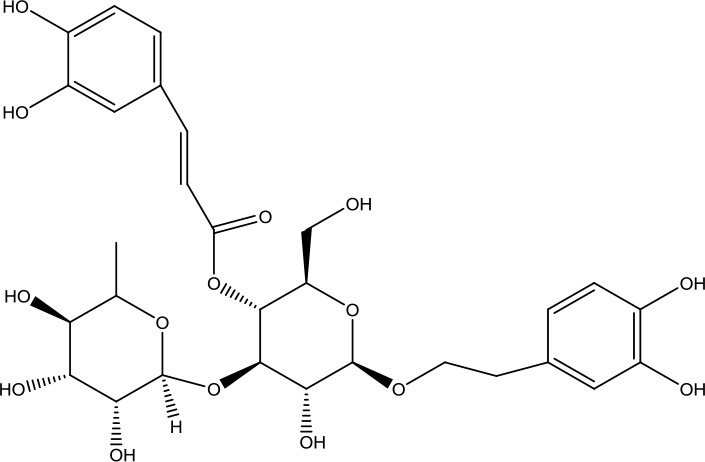	C_29_H_36_O_15_	61276-17-3	Neuroprotective effect; antioxidant effect; immunomodulatory effect	Huang et al., 2012; Deng et al., 2016
*Shanzhuyu* *(Cornus officinalis Siebold & Zucc.)*	Morroniside	406.38	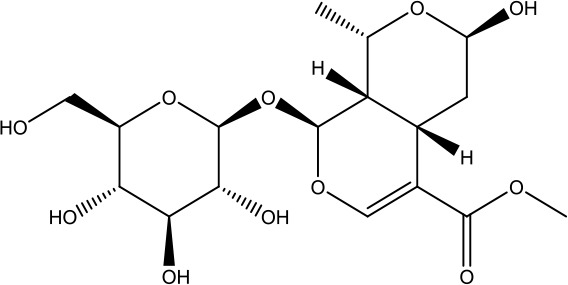	C_17_H_26_O_11_	25406-64-8	Neuroprotective effect; antioxidant effect	Wang et al., 2010; Huang et al., 2018
	Loganin	390.38	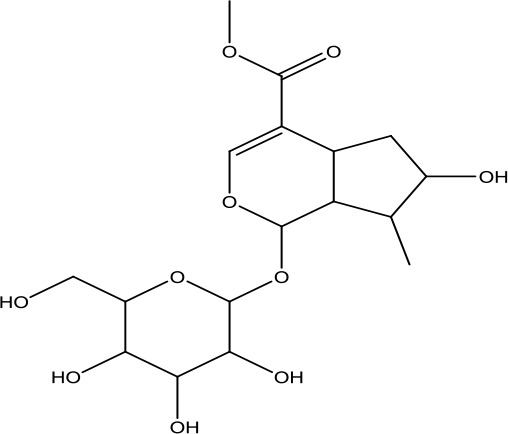	C_17_H_26_O_10_	18524-94-2	Anti-inflammatory; immunomodulatory effect	He et al., 2016; Wang et al., 2018
*Shanyao* *(Dioscorea oppositifolia L.)*	Diosgenin	414.63	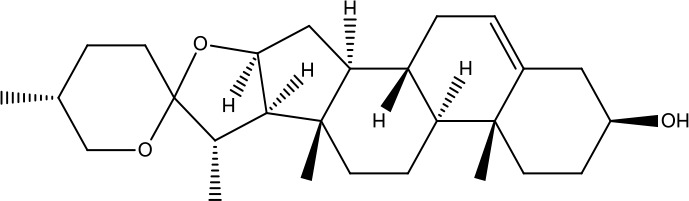	C_27_H_42_O_3_	512-04-9	Anti-tumor; anti-inflammatory; antioxidant effect	Yan et al., 2013; Guo et al., 2016
*Fuling* *(Poria cocos(Schw.) Wolf)*	Pachymic acid	528.76	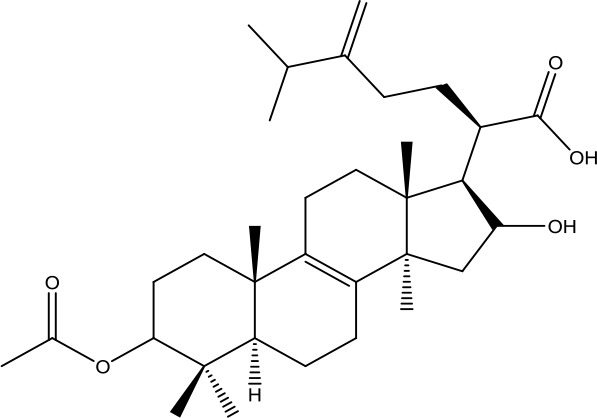	C_33_H_52_O_5_	29070-92-6	Anti-tumor; anti-inflammatory; antioxidant effect; hypoglycemic effect	Li et al., 2017; Qian et al., 2018
	Acetophenone	166.18	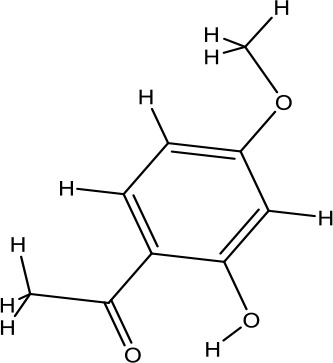	C_9_H_10_O_3_	552-41-0	Anti-inflammatory; anti-allergy; neuroprotective effect; anti-tumor	Sun et al., 2015; Tanaka et al., 2016
*Danpi* *(Paeonia × suffruticosa Andrews)*	Paeoniflorin	480.47	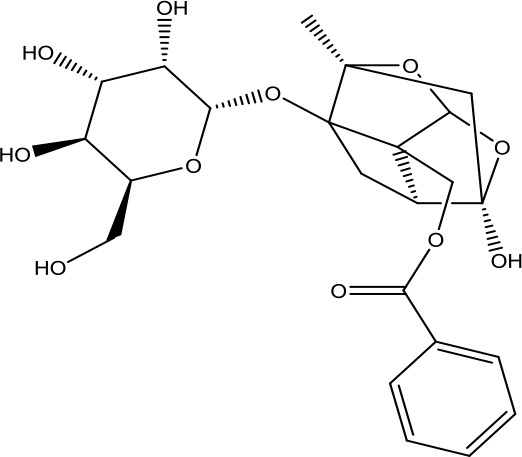	C_23_H_28_O_11_	23180-57-6	Anti-inflammatory; antioxidant effect; antidepressant; enrich the blood	Wu et al., 2009; Zhou et al., 2017
	Alisol A	490.72	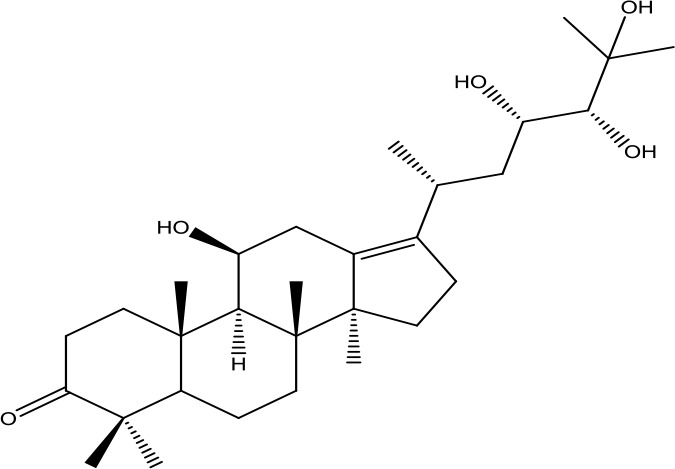	C_30_H_50_O_5_	19885-10-0	Diuretic action; anti-atherosclerosis	Yu et al., 2011; Zhang et al., 2015
*Zexie* *(Alisma plantago-aquatica L.)*	Alisol B	472.70	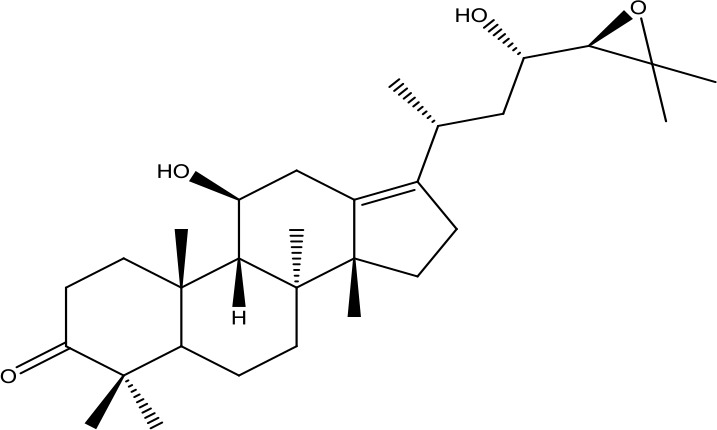	C_30_H_48_O_4_	18649-93-9	Diuretic action;	Gu et al., 2015

### Construction of the Interaction Network and Network Analysis

**Figure 1:**
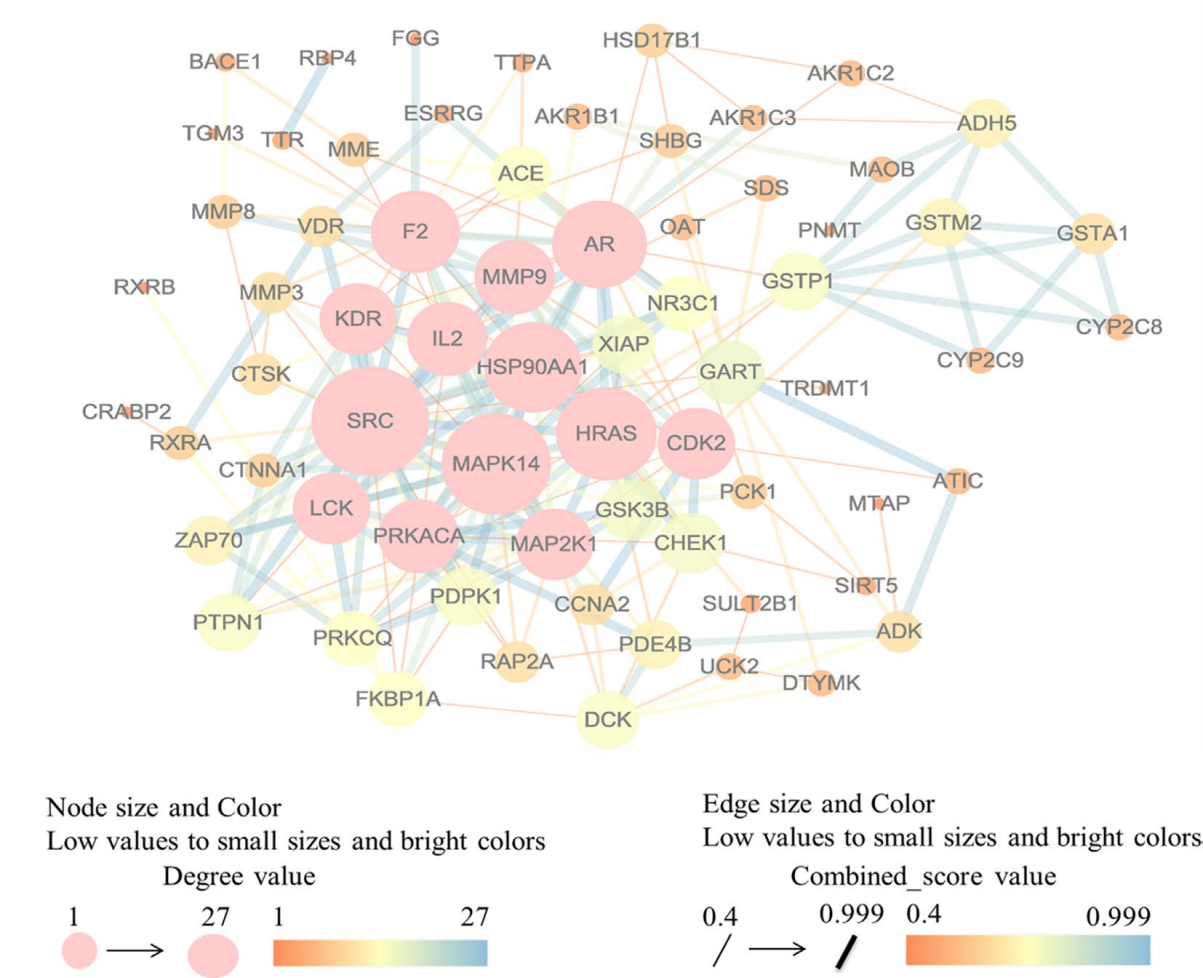
Candidate target genes identified in the protein-protein interaction network constructed using Cytoscape software.

Ranked by fit score in descending order, three hundred potential targets were predicted by PharmMapper. We selected the top 10 targets of each component, if one gene symbol of a component with a different subunit remained. Subsequently, 79 potential targets were selected for further investigation. In the present study, the components of SQW could dock the same or different targets, implying that SQW had a therapeutic effect on the treatment of KYDS through a “multiple components-multiple targets” mechanism. We evaluated 79 potential targets by using the STRING version 10.0 database to identify the interactions between identified 68 proteins. Then, we constructed a PPI network (**Figure 1**) by using Cytoscape. We deleted the isolated pairs of linked nodes, which were not meaningful. The resulting network was composed of 68 nodes and 229 edges, with 27 as the maximum degree of connectivity of a node and 1 as the minimum. We evaluated a node with a degree, which denotes the number of edges between a node and other nodes in a network. A high-degree node was the most influential node in the network, and a hub node was a component of a network with a high-degree node. The average degree of connectivity of the nodes in the network was 6.74, and the standard deviation was 5.84. In this study, we selected the hub nodes with a degree of connectivity set as ≥ the mean value + standard deviation. *SRC* (degree = 27), *MAPK14* (degree = 24), *HRAS* (degree = 21), *HSP90AA1* (degree = 20), *AR* (degree = 19), *F2* (degree = 17), *PRKACA* (degree = 14), *MMP9* (degree = 14), *IL2* (degree = 14), *MAP2K1* (degree = 13), *LCK* (degree = 13), *KDR* (degree = 13), and *CDK2* (degree = 13). The hub nodes maintain the stability of the network and show the occurrence of KYDS, which involves multiple genes and multi-dimensional regulation.

### GO Enrichment and Pathway Analysis for Potential Targets of SQW

**Figure 2:**
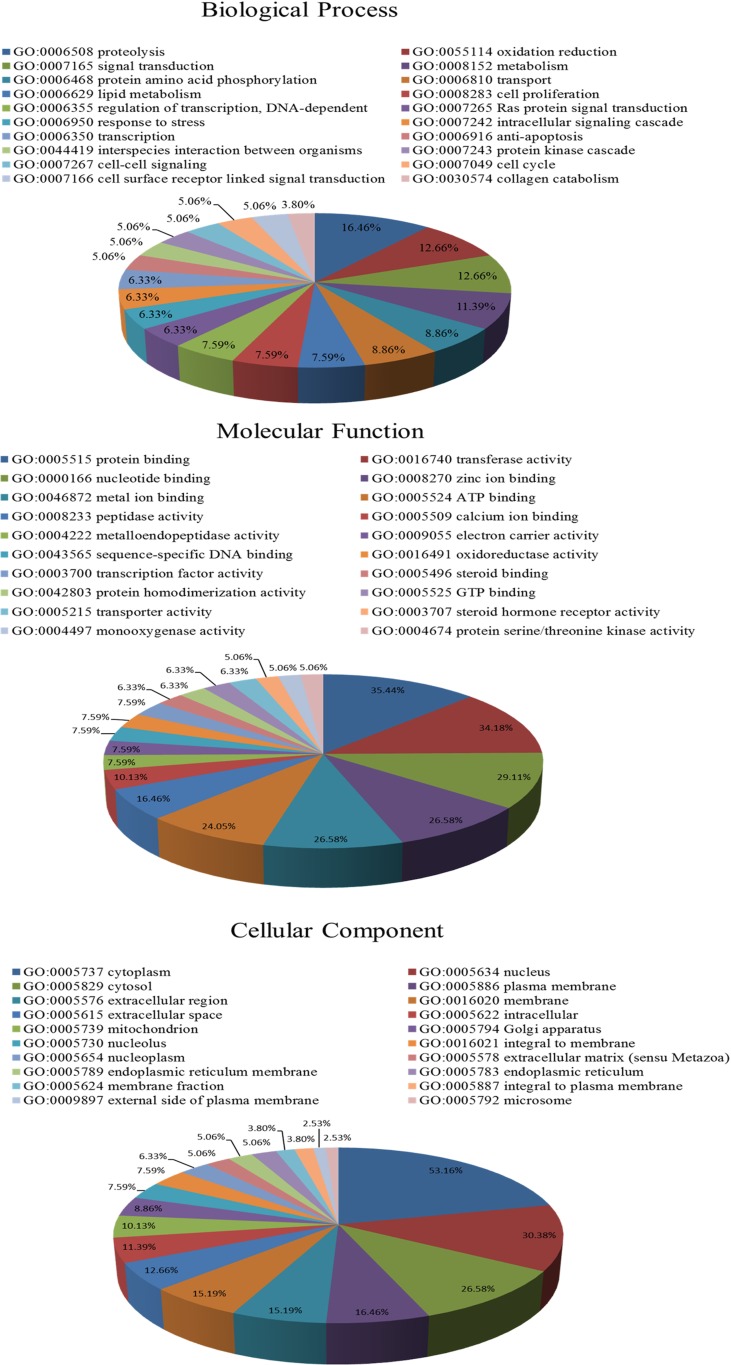
GO enrichment analysis of the potential target genes predicted in the PharmMapper database. **(A)** Biological process enrichment; **(B)** molecular function enrichment; **(C)** cell component enrichment.

We imported the selected potential 79 target genes into the Molecule Annotation System for GO enrichment and pathway analysis. GO analysis results revealed that the functions of these potential targets are related to many biological processes that may be important for the occurrence and development of KYDS, such as proteolysis, oxidation reduction, signal transduction, and metabolism. Binding (protein, nucleotide, zinc ion, metal ion) and activity (transferase, peptidase) are closely related in molecular function and the cellular components, including cytoplasm, nucleus, and cytosol, these proteins were ranked highly as potential targets (**Figure 2**).

**Figure 3:**
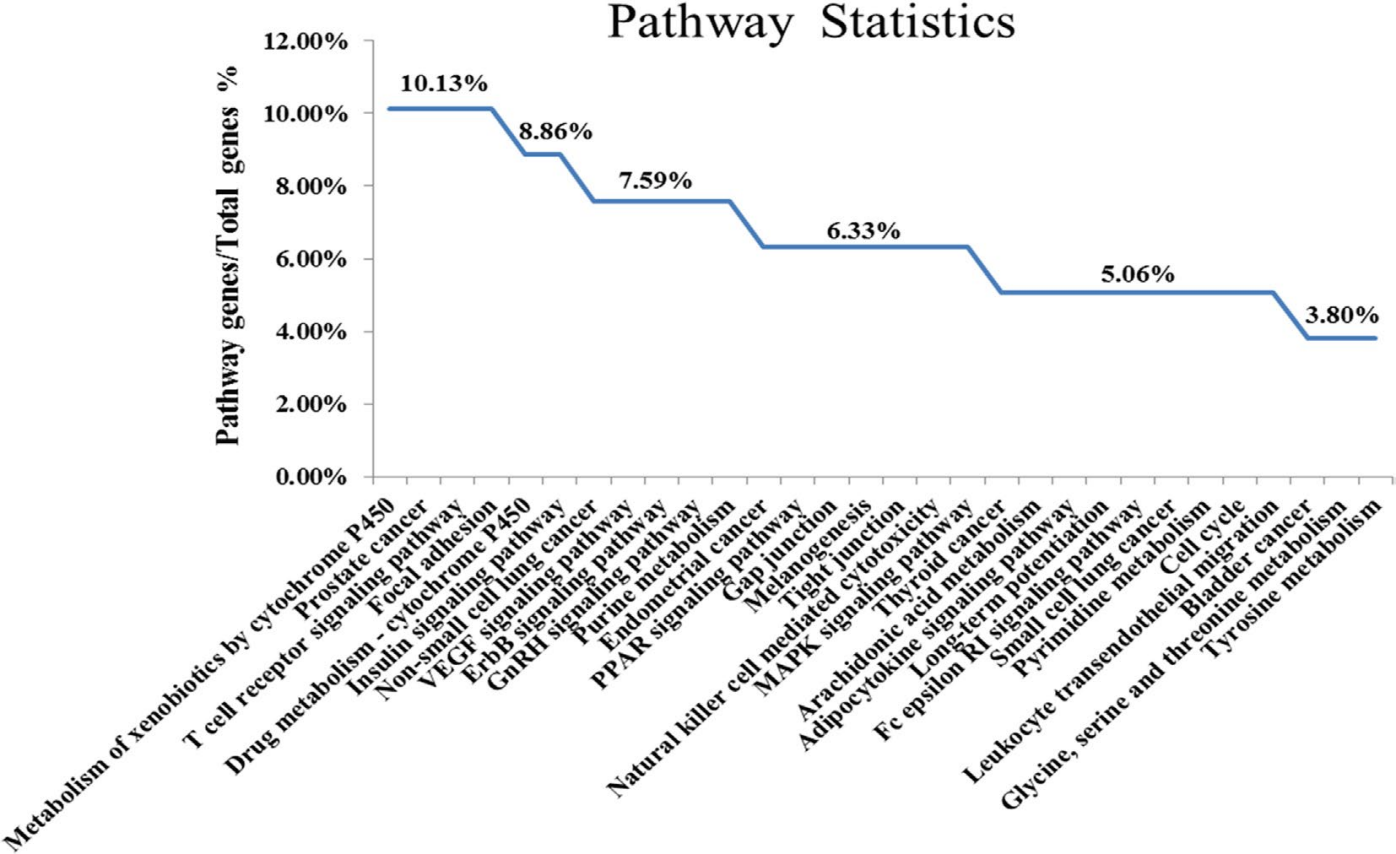
KEGG pathway enrichment of the potential target genes in SQW (top 30).

A total of 105 pathways were obtained by GO analysis, from which we selected the top 76 pathways that met the criterion of *p* < 0.05. Numerous pathways for potential target genes were identified. Our study found that the ErbB signaling pathway, VEGF signaling pathway, and MAPK signaling pathway are associated with signal transduction, the insulin signaling pathway, metabolism of xenobiotics by cytochrome P450, drug metabolism—cytochrome P450, and the PPAR signaling pathway. Androgen and estrogen metabolism are associated with the endocrine system. The focal adhesion and the T cell receptor signaling pathway are also closely related to immunological stress or inflammation. Moreover, we found some disease-related pathways such as prostate cancer, non-small cell lung cancer, endometrial cancer, and thyroid cancer, which indicate that SQW has a potential application in other diseases (**Figure 3**). The results prompted that SQW ameliorated the imbalance of body by regulating the neurological, endocrine, and immune processes.

### Pharmacology Network of SQW

**Figure 4:**
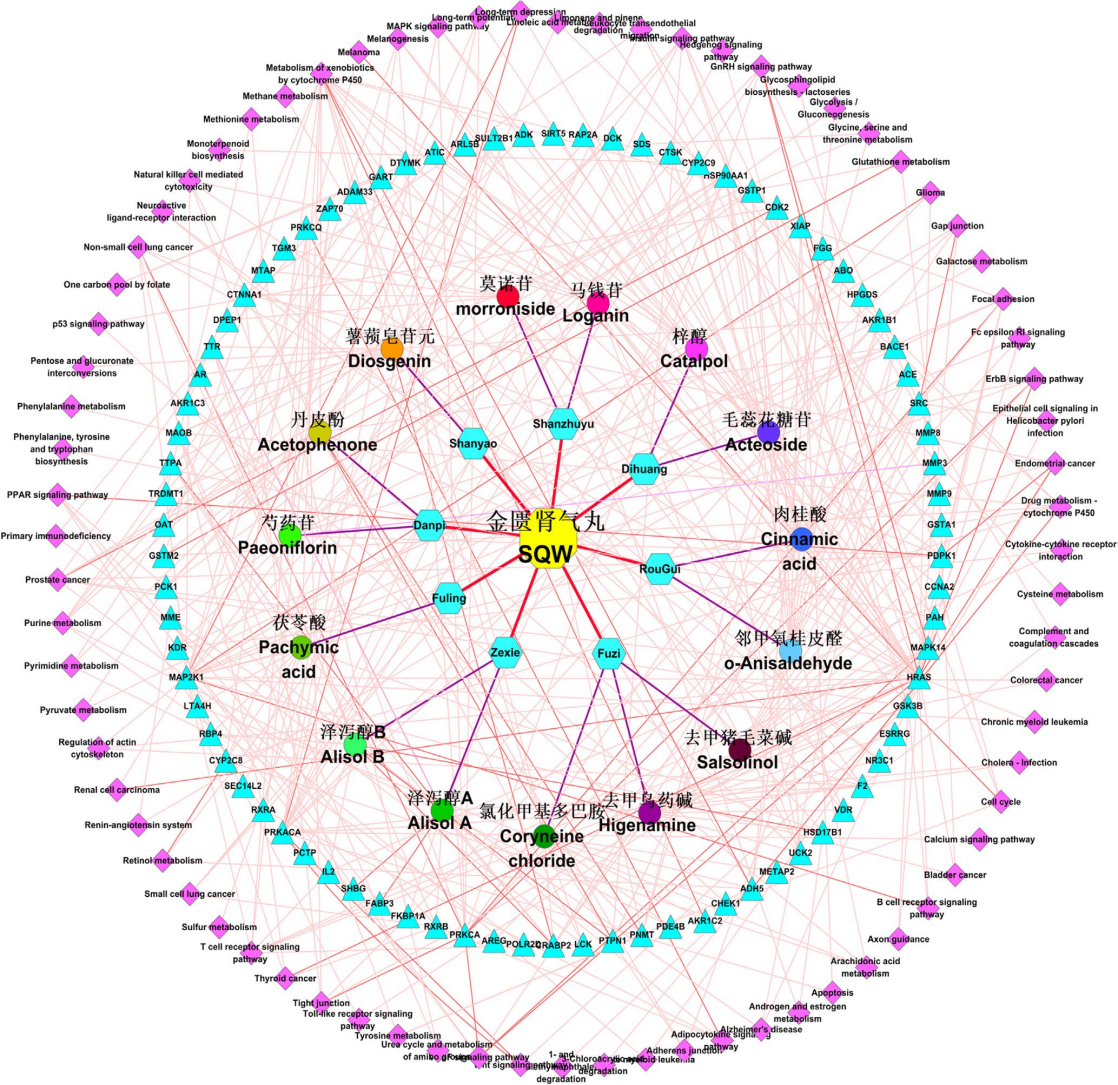
Pharmacology network of the “components-targets-pathways” regulated by SQW (pink. diamonds indicate pathways, cerulean triangles indicate targets, different colored circles indicate the chemical components, cerulean hexagons indicate the herbs in SQW, and the yellow octagon represents SQW. Red lines represent the relation of herbs with SQW; purple lines indicate the relation of herbs between its chemical components; and light pink lines indicate the relation of chemical components, component targets, and component pathways).

We constructed a pharmacology network of SQW (**Figure 4**) using the Cytoscape software, which showed the relationships among the constituents, chemical components, and potential targets of SQW and the selected 76 pathways (*p* < 0.05). We obtained a preliminary understanding of the mechanism of SQW through this network. The potential targets of the effective components are distributed in different metabolic pathways to jointly affect the occurrence and development of KYDS.

### Adenine-Induced KYDS

**Figure 5:**
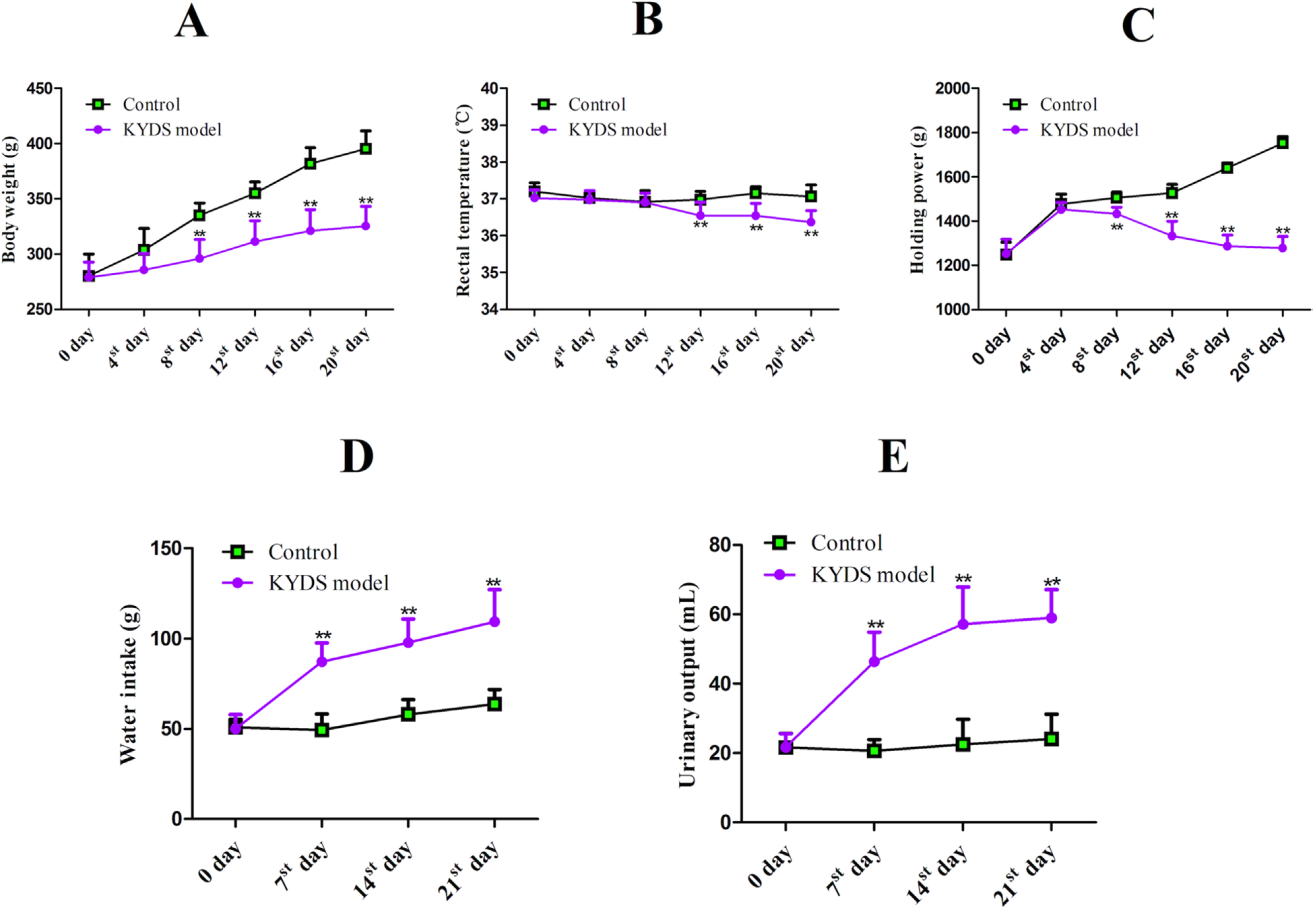
The effect of adenine on body weight **(A)**, rectal temperature **(B)**, holding power **(C)**, water intake **(D)**, and urinary output **(E)** in rats. **p < 0.01, the KYDS model group (n = 20) versus the control group (n = 10). Values are presented as mean values ± SD. The *p*-values were calculated using a one-way ANOVA.

To validate the establishment of the animal model, the body weight, rectal temperature, and the holding power were measured on days 0, 4, 8, 12, 16, and 20. The results (**Figure 5A, B, and C**) demonstrated that the body weight, temperature, and the holding power values of the KYDS model rats gradually decreased as the time increased compared to those of the rats in the control groups (*p* < 0.01), whereas the water intake and urinary output of KYDS model rats were higher than those of the rats in the control groups (*p* < 0.01) on the 7th, 14th, and 20th days (**Figure 5D and E**). As shown in **Table 3**, the contents of BUN, Scr, ACTH, and CORT were determined in rat serum on the 21^st^ day. The BUN and Scr of the KYDS model were significantly increased (*p* < 0.01), whereas the ACTH and CORT of the KYDS model rats were significantly lower than those of the rats in the control group (*p* < 0.01). Moreover, the concentration of 17-OHCS in the KYDS rats was decreased compared with that in the control group rats (*p* < 0.01), but the U-TP in the urine of KYDS model rats was significantly increased (*p* < 0.01). These results indicated that the rats presented symptoms such as sluggishness, languorousness, and a crouched posture, which are the typical pathological features of KYDS. The biochemical results indicated that the KYDS model was successfully established for subsequent experiments.

**Table 3:** Effects of adenine on BUN, Scr, ACTH, CORT, U-TP, and 17-OHCS in rats.

Groups	BUN (mmol/l)	Scr (µmol/l)	ACTH (ng/ml)	CORT (ng/ml)	U-TP (mg/24 h)	17-OHCS (nmol/l)
Control	6.12 ± 0.95	58.70 ± 2.50	88.93 ± 6.43	64.89 ± 6.57	21.72 ± 5.90	29.20 ± 9.62
KYDS model	45.55 ± 13.18**	223.00 ± 64.19**	74.26 ± 16.80**	41.87 ± 18.20**	30.27 ± 3.53**	22.27 ± 3.53**

The values are presented as the means ± SD. **p < 0.01 KYDS model group (n = 20) compared with control group (n = 10).

### Treatment of KYDS With SQW

**Figure 6:**
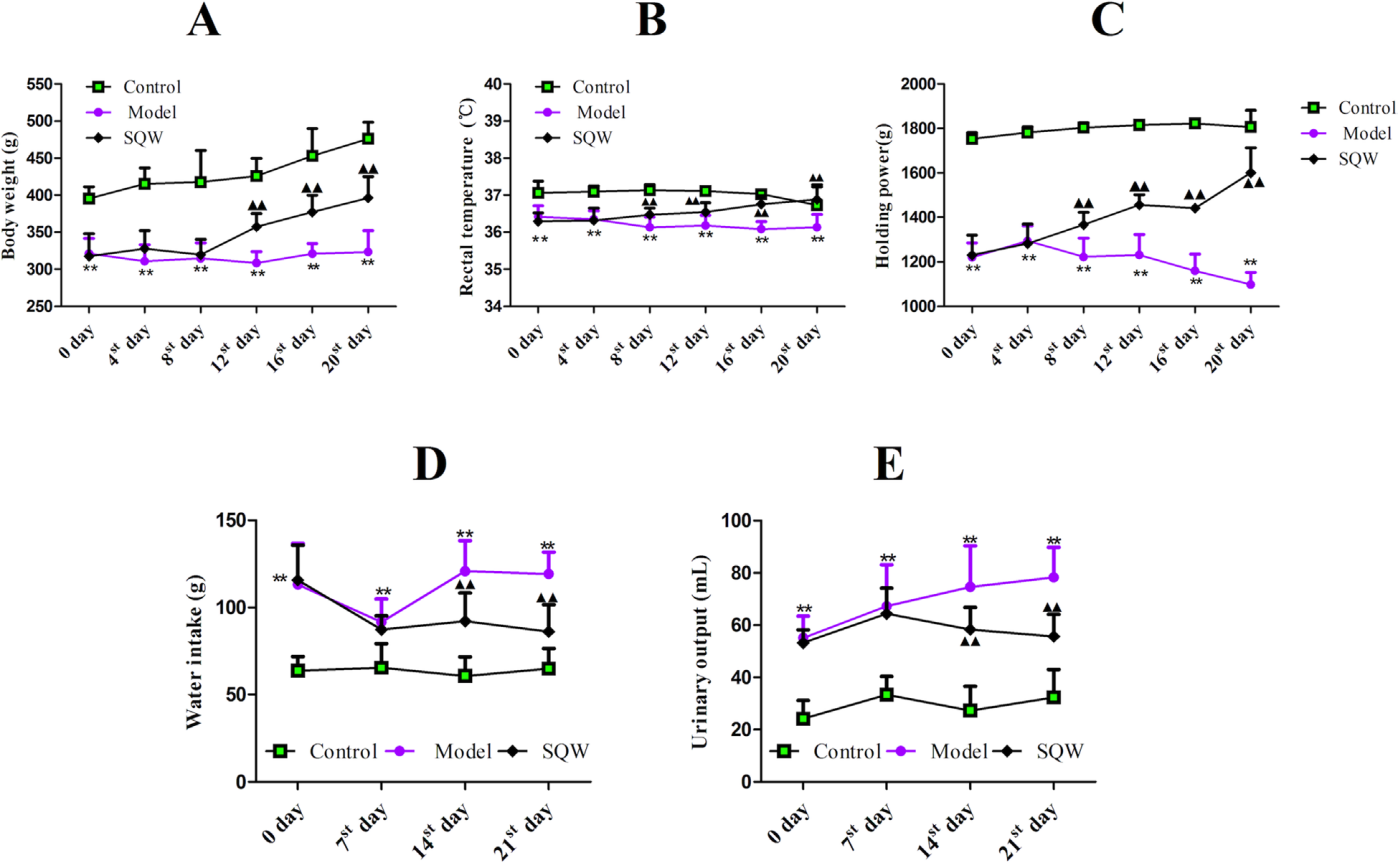
Effects of SQW on body weight **(A)**, rectal temperature **(B)**, holding power **(C)**, water intake **(D)**, and urinary output **(E)** in rats. **p < 0.01, the model group (n = 10) versus the control group (n = 10). ▲▲ *p* < 0.01, the SQW group (n = 10) versus the model group (n = 10). Values are presented as the means ± SD. The *p*-values were calculated using a one-way ANOVA.

Treatment group rats recuperated after intra-gastric administration of SQW. The body weight improved significantly in the SQW-treated rats compared to that in model group rats (*p* < 0.01) on the 12^th^ day of intra-gastric administration of SQW (**Figure 6A**). The rectal temperature and holding power of the SQW-treated rats were ameliorated compared with that in the model groups (*p* < 0.01) at the beginning of the 8^th^ day of intra-gastric administration of SQW (**Figure 6B and C**). The levels of water intake and urinary output in the SQW-treated rats gradually returned to the baseline levels (*p* < 0.01) of the control group (**Figure 6D and E**). The body weight, rectal temperature, holding power, water intake, and urinary output of the model groups showed significant differences compared with those in the control groups (*p* < 0.01) throughout the treatment period. As shown in **Table 4**, the SQW treatment obviously improved the numeral values of ACTH, CORT, and 17-OHCS (*p* < 0.01), while BUN, Scr, and U-TP showed a significant decrease compared with the model groups (*p* < 0.01), demonstrating that SQW could effectively ameliorate KYDS and had a therapeutic effect on the rat KYDS models.

**Table 4:** Effects of SQW treatment on BUN, Scr, ACTH, CORT, U-TP, and 17-OHCS in rats.

Groups	BUN (mmol/l)	Scr (µmol/l)	ACTH (ng/ml)	CORT (ng/ml)	U-TP (mg/24 h)	17-OHCS (nmol/l)
Control	5.69 ± 0.61	53.50 ± 4.18	101.11 ± 6.57	82.15 ± 8.47	28.73 ± 6.51	45.31 ± 3.62
Model	34.37 ± 12.99**	179.79 ± 47.52**	87.04 ± 5.07**	38.75 ± 5.96**	51.58 ± 8.59**	32.01 ± 2.40**
SQW	10.41 ± 3.02^▲▲^	67.21 ± 13.82^▲▲^	99.00 ± 3.56^▲▲^	55.51 ± 4.35^▲▲^	27.86 ± 5.81^▲▲^	41.19 ± 3.01^▲▲^

The values are presented as mean values ± SD. **p < 0.01 compared with the control group (n = 10); *▲▲* p < 0.01 SQW group (n = 10) compared with the model group (n = 10).

#### Results of qPCR for Candidate Target Genes

**Figure 7:**
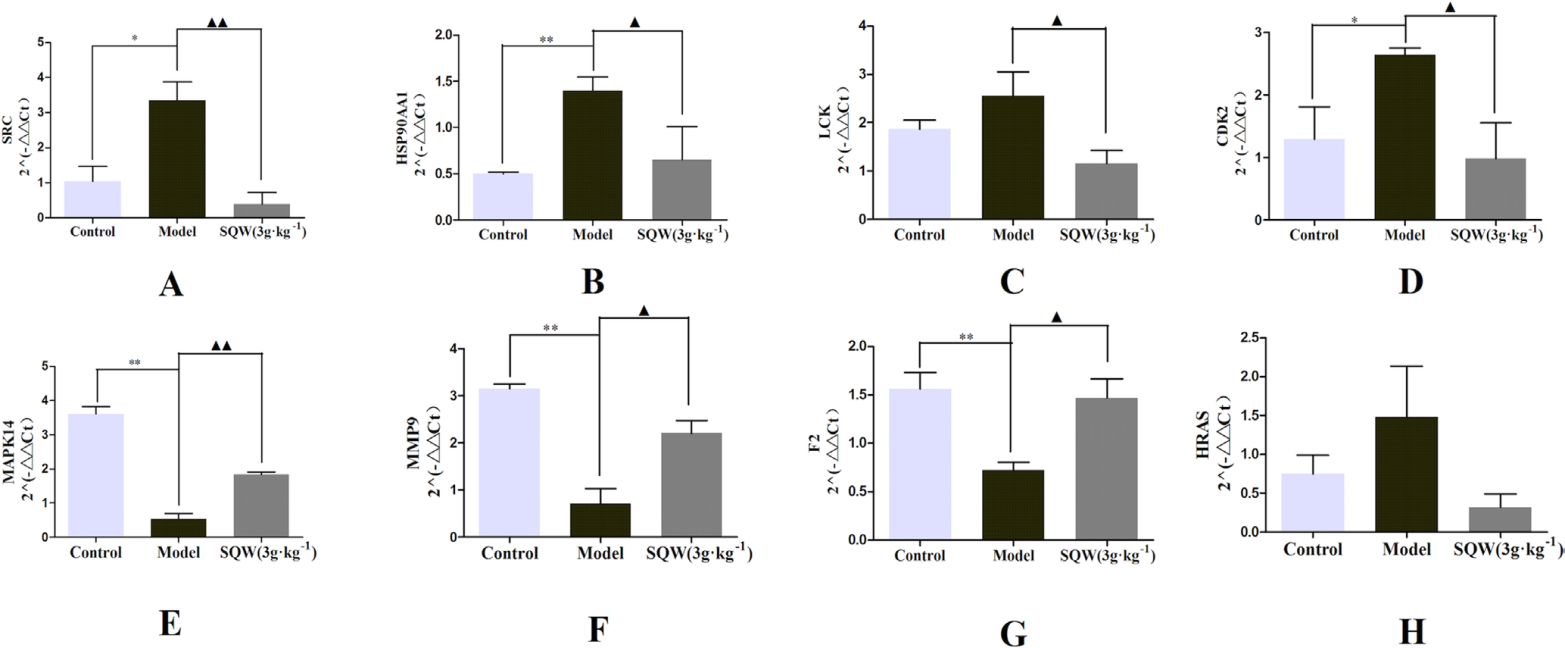
The effects of the control, KYDS model, and SQW treatments (3 g/kg/day) on SRC **(A)**, HSP90AA1 **(B)**, LCK **(C)**, CDK2 **(D)**, MAPK14 **(E)**, MMP9 **(F)**, F2 **(G)**, HRAS **(H)** mRNA expression in the kidney. Values are expressed as mean values ± SD. **p < 0.01 or *p < 0.05 compared with the control group; ▲▲ *p* < 0.01 or ▲ *p* < 0.05 compared with the model group.

The hub genes were identified in the PPI network with high degree of connectivity. Among them, *SRC, MAPK14, HRAS, HSP90AA1, F2, LCK, CDK2*, and *MMP9* were closely related to the emperor’s constituents. Then, we explored the effect of SQW on mRNA expression in the kidney using qPCR, as shown in **Figure 7**. The mRNA expression levels of *SRC, HSP90AA1, LCK*, and *CDK2* in the SQW-treated group were significantly decreased compared to those in the model group (**Figure 7A–D**), whereas *MAPK14, MMP9*, and *F2* expression levels were significantly higher in the SQW-treated group than those in the model group (**Figure 7E–G**). Although the mRNA expression levels of *HRAS* showed no significant difference compared with the model group, there was a weak trend (**Figure 7H**). These results indicate that SQW treatment could effectively ameliorate KYDS *via* the synergy of multiple targets.

### Results of Ultra-Performance Liquid Chromatography

**Figure 8:**
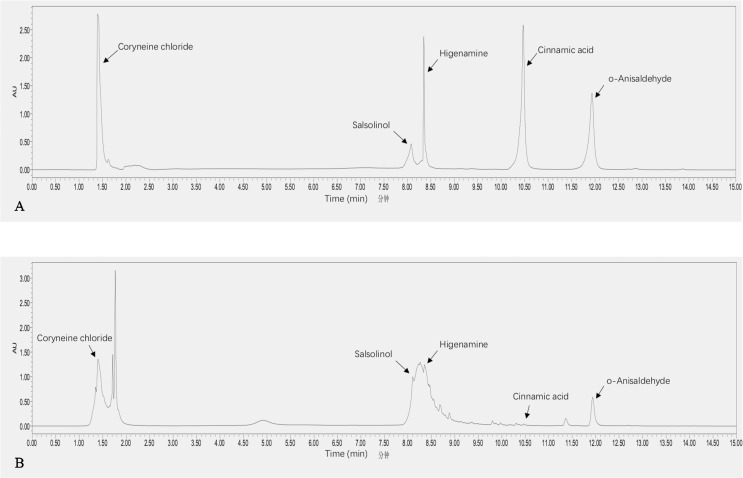
The performances of higenamine, coryneine chloride, salsolinol, o-anisaldehyde, and cinnamic acid in SQW (40 mg/ml) **(B)** and in mixed standards (2 mg/ml) **(A)**. The UV detection wavelength was at 208 nmOs.

The main five components (higenamine, coryneine chloride, salsolinol, o-anisaldehyde, and cinnamic acid), which belong to the *jun* herbs *Ramulus Cinnamomi* and *Radix aconiti lateralis preparata*, were detected by UPLC in SQW (**Figure 8**). The gradient program was as follow: 0–5 min, 0.5–2% A; 5–6 min, 2–30% A; 6–8 min, 30–50% A; 10–12 min, 50–70% A; 12–15 min, 70–0.5% A. The UV detection wavelength was at 208 nm. However, the peak of cinnamic acid seemed to be weak.

## Discussion

KYDS is most prevalent in older men and women and increases with age (Chen et al., 2010; Rong et al., 2016). TCM considers KYDS to be a complex kidney disorder, and “kidney *yang*” activates the power of human vitality (Lu et al., 2011; Huang et al., 2013; Zhao et al., 2013). The primary cause of KYDS is a decline in kidney-*yang* and transformative action, which is similar to a debilitating disease, such as chronic prostatitis, nephrotic syndrome, adrenocortical insufficiency, chronic nephritis, and diabetes mellitus in Western medicine. SQW is a typical TCM formula widely used for the treatment of chronic diseases associated with KYDS in China. Nevertheless, due to the complex pathological properties and multiple targets in KYDS, it is not easy to explore the mechanism of action of SQW using traditional methods. In this study, reverse pharmacophore docking and network pharmacology strategies were used to study the characteristics of “multiple components-multiple targets-multiple pathways” associated with SQW in the treatment of KYDS.

PharmMapper was designed to identify potential target candidates for given small molecules (drugs, natural products, or other newly discovered compounds with unidentified binding targets) by the mutual recognition of space and the ability to find the best mapping configurations (Liu et al., 2010). Ma et al. (2016) showed the “multiple components-multiple targets-multiple pathways” mechanism of *Naoxintong* capsule with the PharmMapper database and network pharmacology. Tao et al. (2016) used PharmMapper and the KEGG bioinformatics websites to predict the target proteins and related pathways of *Chuanbei Pipa* dropping pills to clarify the anti-inflammatory and cough-suppressing mechanisms. Using a network pharmacology method provides a basis for understanding the mechanism of action of SQW and is indispensable in the study of complex drugs.

We successfully predicted the drug targets of 15 compounds in SQW. The results of PPI network suggested 13 hub genes, which play important roles in the PPI. Among them, *SRC, MAPK14, HRAS, HSP90AA1, F2, LCK, CDK2*, and *MMP9* were closely associated with higenamine, coryneine chloride, salsolinol, o-anisaldehyde, and cinnamic acid. These compounds were found in *R. Cinnamomi* and *Radix aconiti lateralis preparata*, the *jun* herb of SQW treating the main cause or primary symptoms of KYDS.

Proto-oncogene tyrosine-protein kinase Src (*SRC*) is a non-receptor tyrosine kinase. Once *SRC* is activated, the intracellular signal transduction cascades are triggered and subsequently multiple cellular functions such as cell proliferation, differentiation, and metabolism are modulated. Further investigations revealed that *SRC* activation is critically involved in the development of chronic kidney disease. Yan et al. (2016) observed that *SRC* kinase is activated in cultured kidney fibroblasts, and the inhibition of *SRC* by PP1, a selective small-molecule inhibitor of *SRC* kinase, appeared to disrupt TGFβ1/Smad3 and epidermal growth factor receptor (EGFR) signaling. Another study demonstrated that the inhibitor of SRC kinase effectively blocked the expression of α-SMA, which is associated with the progression of renal fibrogenesis (Hu et al., 2014). Even more, *SRC* can be activated by autophosphorylation of Tyr416, which is induced in response to a wide variety of cytokines/growth factors/transmembrane receptor proteins, including receptor tyrosine kinases, cytokine receptors, TGF-β1, and EGF (Yan et al., 2016; Zhou and Liu, 2016). Thus, *SRC* may be a potential therapeutic target for the treatment of chronic renal fibrosis with KYDS.

Mitogen-activated protein kinase 14 (*MAPK14*) encodes P38 mitogen-activated protein kinase and can be activated by various environmental stressors and pro-inflammatory cytokines (Han et al., 2015). *MAPK14* regulates the activation of several transcription factors responses, including gene expression, growth, inflammation, metabolism, and apoptosis (Umasuthan et al., 2015). *MAPK14* activity-deficient mice had less kidney dysfunction, inflammation, and apoptosis in acute folate nephropathy, while *MAPK14* siRNA targeting decreased inflammation and cell death in cultured tubular cells (Ortiz et al., 2017). We conclude that *MAPK14* promoted kidney injury through the promotion of inflammation and cell death and that it is a putative novel therapeutic target of SQW to ameliorate KYDS.

*HRAS*, a small GTPase from the Ras family, encodes the GTPase HRas, which is also known as the transforming protein p21 (Sugita et al., 2018). *HRAS* plays a role in regulating the growth, differentiation, and death of endothelial cells while enhancing the effects of the growth factor (Burgoyne et al., 2012). Moreover, *HRAS* participates in focal adhesion and the MAPK pathway by relieving inflammation (Tao et al., 2016).

Heat shock protein (HSP) is a highly conserved protein that is synthesized in response to physical, chemical, biological, and/or mental stimulation. Heat shock protein HSP 90α (*HSP90AA1*) belongs to the HSP90 protein superfamily, which is a molecular chaperone of numerous oncoproteins and a mediator of cellular homeostasis to maintain cell survival under stimulation (Trepel et al., 2010). Hsp 90 inhibition represses the TLR4-mediated NF-κB activity primarily through IKK to reduce renal ischemia-reperfusion acute injury (O’Neill et al., 2015). Moreover, inhibiting HSP90 activation prevents the development of renal fibrosis through the degradation of TβRII depending on Smurf2-mediation (Noh et al., 2012). These intriguing findings suggest that the kidney-protective functions of SQW may occur by regulating the expression of HSP90AA1.

Coagulation factor II (*F2*) encodes the prothrombin protein, which functions in blood homeostasis, inflammation, and wound healing. *Qi* deficiency and blood stasis are the key factors of KYDS, which is characterized by decreased gasification, a disorder of vital energy and blood, and cold limbs. We speculate that blood rheology abnormalities cause the deficiency of heat production and the ability of the kidney-yang to transfer body fluid into energy. However, the function of *F2* in KYDS should be further studied.

Tubular epithelial cells (TECs) play an important role in renal diseases, especially in tubulointerstitial inflammation and fibrosis, which is a pathological process involved in a variety of cytokines and inflammatory mediators. Lymphocyte-specific protein-tyrosine kinase (*LCK*) and a SRC family protein-tyrosine kinase are located in the cytoplasm of TECs and form the key signal transduction molecule in the process of intracellular signal transduction (Singh et al., 2017). Li et al. have investigated the effect of the LCK pathway activation on the IL-12 signal transduction of TECs and found that *LCK* may regulate the LCK c-Jun signaling pathways in TEC, while the inflammation of TEC mediated by the activation of the LCK pathway is related to the expression of c-Jun promoted by IL-12 (Li et al., 2001).

Glomerular mesangial cell proliferation is a common pathological feature of many glomerular diseases (Lin et al., 2017). Cyclin-dependent kinase 2 (*CDK2*) is a serine/threonine-protein kinase involved in the control of the cell cycle. Yu et al. (2007) observed that the proliferation of mesangial cells is directly related to the high expression of *CDK2*, which indicates that SQW probably improves KYDS by depressing the expression of *CDK2*.

Renal fibrosis is a common disease with pathological characteristics of the accumulation of extracellular matrix (ECM) and also strongly associated with the progression of chronic kidney disease to end-stage renal disease. Matrix metalloproteinases (MMPs) are renal, physiological regulators of ECM degradation. Matrix metalloproteinase 9 (*MMP*9), a 92 kDa type IV collagenase, can specifically degrade type IV and V collagens and gelatin to maintain homeostasis of the ECM in the kidney (Lenz et al., 2000). ECM components accumulate due to an imbalance in ECM production and defective ECM degradation by proteolytic enzymes during renal fibrosis (Tsai et al., 2012). The results of GO enrichment showed that protein hydrolysis has an important role, which is consistent with the function of *MMP9*. The preliminary results in our research demonstrated that the medicated serum of 3.0 and 6.0 g/kg SQW significantly increased the expression of MMP9 protein in NRK-52E cells. Target prediction also showed that salsolinol is associated with *MMP9, MMP3*, and *MMP8*, suggesting an interaction relationship between SQW and the matrix metalloproteinases (MMPs) family. These findings suggest that SQW reduces the accumulation of EMC in renal epithelial cells *via* the metalloproteinases.

Moreover, the effect of SQW on AQPs, the aquaporin channel family, and on the relation between *AQP1* and MMP9 showed a trend of enhancement to promote the migration of renal TECs for renal injury repair. Moreover, SQW has a therapeutic effect on water metabolism disorder by promoting the mRNA and protein expression levels of *AQP2*. Furthermore, SQW significantly increased the ACTH, while CORT regulated the hypothalamic-pituitary-adrenal axis to exploit the *R. Cinnamomi* and *Radix aconiti lateralis preparata* role in tonifying the kidney yang (Xu et al., 2014). These results are consistent with the therapeutic effect of SQW observed in the present paper.

Our study showed that SQW treatment dramatically improved the common physiological symptoms of KYDS and had protective effects on the hypothalamic-pituitary-adrenal axis in KYDS model rats. The potential targets of SQW were identified using PharmMapper, bioinformatics, and PPI network analysis. We found 79 potential target genes and identified *SRC, MAPK14, HRAS, HSP90AA1, F2, LCK, CDK2*, and *MMP9* as the key potential therapeutic targets of SQW. The 79 target genes mainly related to the metabolism of xenobiotics by cytochrome P450, prostate cancer, and the T cell receptor signaling pathway. Wang et al. (2016) also reported 14 important potential targets associated with the aldosterone-regulated sodium reabsorption and adrenergic signaling pathways. However, further studies are required to confirm the results of this study. We explored the potential targets and pathways of SQW from a different perspective and using novel methods, and we conclude that multiple components, multiple targets, and multiple pathways of SQW led to a therapeutic effect on KYDS. This study shows that cell proliferation, differentiation, apoptosis, migration (*SRC, HRAS, HSP90AA1, CDK2*), and ameliorating chronic kidney disease (*MAPK14, F2, LCK, MMP9*) appear to play important roles in the therapeutic effect of SQW.

In this study, SQW ameliorated KYDS characteristics in rats presumably by eight target genes. Further studies are needed to analyze the protein levels of these targets. Moreover, other species should be considered for further verification.

## Conclusion

In summary, SQW has a therapeutic effect on the treatment of KYDS through the “multiple components-multiple targets-multiple pathways” mechanism. We found that *SRC, MAPK14, HRAS, HSP90AA1, F2, LCK, CDK2*, and *MMP9* genes were highly involved and may be potential targets in the treatment of KYDS.

## Data Availability Statement

The raw data supporting the conclusions of this manuscript will be made available by the authors, without undue reservation, to any qualified researcher.

## Ethics Statement

This study was carried out in accordance with the recommendations of the Ethics of Committee of Zhejiang Chinese Medical University (permit number: SYXK 2013-0115). The protocol was approved by the Ethics of Committee of Zhejiang Chinese Medical University (permit number: SYXK 2013-0115). All procedures were performed under sodium pentobarbital anesthesia, and all efforts were made to minimize suffering.

## Author Contributions

JZ, CH, YY and CL conceived and designed the experiments. JZ and CH performed the experiments. HC, XZ and YZ analyzed the data. JZ, HC, TE, YY and CL contributed reagents/materials/analysis tools. JZ and TE wrote and edited the paper. JZ and CH contributed equally to this work. TE, YY and CL contributed equally to this work.

## Funding

This study was supported by grants from the National Natural Science Foundation of China (Nos. 81673839 and 81373507), the Project of National Great New Drug Research and Development (No. 2012ZX09503001-001), and Science and Technology Innovation Project of Zhejiang Province College Students (Grant No. 2017R410049) and Zhejiang Province Administration of Traditional Chinese Medicine (no. 2015ZA073). The funders had no role in the study design, data collection and analysis, decision to publish, or preparation of the manuscript.

## Conflict of Interest Statement

The authors declare that the research was conducted in the absence of any commercial or financial relationships that could be construed as a potential conflict of interest.

## Abbreviations

17-OHCS, 17-hydroxy-corticosteroid; ACTH, adrenocorticotrophic hormone; AQP, aquaporin; BUN, blood urea nitrogen; CAS, Chemical Abstract Service; *CDK2*, cell division protein kinase 2; CNKI, China National Knowledge Infrastructure; CORT, cortisol; ECM, extracellular matrix; *F2*, prothrombin; GO, Gene Ontology; HPLC, high performance liquid chromatography; *HRAS*, GTPase HRas; *HSP90AA1*, heat shock protein HSP 90α; KEGG, Kyoto Encyclopedia of Genes and Genomes; KYDS, kidney *yang* deficiency syndrome; *LCK*, Lymphocyte-specific protein-tyrosine kinase LCK; *MAPK14*, mitogen-activated protein kinase 14; *MMP9*, matrix metalloproteinase-9; PPI, protein–protein interaction; Scr, serum creatinine; SQW, *Shen Qi Wan*; *SRC*, proto-oncogene tyrosine-protein kinase SRC; STRING, (Search Tool for the Retrieval of Interacting Genes/Proteins; TCM, traditional Chinese medicine; TcmSP™, Traditional Chinese Medicine Systems Pharmacology Database; UPLC, ultra-performance liquid chromatography; U-TP, urine total protein.
